# A revision of the pelomedusoid turtle *Jainemys pisdurensis* from the Late Cretaceous (Maastrichtian) Lameta Formation of India

**DOI:** 10.7717/peerj.9330

**Published:** 2020-06-22

**Authors:** Walter G. Joyce, Saswati Bandyopadhyay

**Affiliations:** 1 Department of Geosciences, University of Fribourg, Fribourg, Switzerland; 2 Geological Studies Unit, Indian Statistical Institute, Kolkata, India

**Keywords:** India, Maharashtra, Cretaceous, Maastrichtian, Testudines, Pleurodira, Bothremydidae, Kurmademydini, Systematics, Paleobiogeography

## Abstract

**Background:**

*Jainemys pisdurensis* comb. nov. is an extinct pleurodiran turtle from the Late Cretaceous (Maastrichtian) of India, previously referred to *Carteremys* and *Shweboemys*. The holotype, an eroded skull, had been collected near the village of Pisdura, south of Nagpur, in Maharashtra State, while all referred shell material originates from coeval sediments exposed at the nearby village of Dongargaon. Initial estimates believed this turtle to either be an early representative of Podocnemididae or a basal representative of Pelomedusoides.

**Methods:**

We here figure and describe all specimens that had previously been referred to *Jainemys pisdurensis* comb. nov. We furthermore re-evaluate the validity of this fossil turtle and explore its phylogenetic relationships within Pleurodira.

**Results:**

The holotype of *Jainemys pisdurensis* comb. nov. displays a morphology that differs substantially from that originally reported. Most notably, the palatines only have a minor contribution to the broad triturating surfaces but have a broad midline contact with each other, the pterygoids only have a midline contact of intermediate length and do not contact the opisthotics posteriorly, the basisphenoid is broad and short, and the opisthotics do not contribute to the flooring of the cavum acustico-jugulare. The referred shell material also displays a morphology different from that reported originally, in particular in that vertebral I does not contribute to the anterior margin of the carapace while the nuchal does. Phylogenetic analysis places the cranial material within the bothremydid clade Kurmademydini, while the shell material is placed in an unresolved polytomy at the base of this clade. *Jainemys pisdurensis* is confirmed to be a valid species of pleurodiran turtle, but the high diversity of coeval kurmademydines in India demands removal of the postcranial remains from this taxon. The realization that all valid species of Late Cretaceous (Maastrichtian) turtles from India form a clade supports the hypothesis that India was physically separated from the rest of Gondwana at this time.

## Introduction

*Jainemys pisdurensis* comb. nov. is a poorly understood fossil turtle from the Late Cretaceous of India (Jain, 1977). The species was originally described by Jain (1977) based on a single, eroded skull that had been collected near the village of Pisdura in eastern Maharashtra in outcrops of the Maastrichtian Lameta Formation. As the skull from Pisdura seemed to resemble the holotype of *Carteremys leithii* (Carter, 1852) in having a deep upper temporal emargination, Jain (1977) referred his new turtle to *Carteremys* to form *Carteremys pisdurensis*. Wood (1985) soon after suggested based on the description of Jain (1977) that this turtle may be a representative of the podocnemidid *Shweboemys*. A year later, Jain (1986) referred new shell material and associated limb and girdle fragments to this species from coeval sediments exposed at Dongargaon, about 20 km south of Pisdura, likely based on temporal and spatial considerations. Jain (1986) furthermore followed Wood (1985) by proposing the new combination *Shweboemys pisdurensis*. In a series of papers pertaining to the cranial morphology of pelomedusoid turtles, Gaffney, Chatterjee & Rudra (2001) and Gaffney et al. (2003) initially confirmed assignment of the Pisdura turtle to *Shweboemys*, while Gaffney, Tong & Meylan (2006) highlighted that it certainly does not represent a bothremydid. More recently, however, Gaffney et al. (2011) noted that the Pisdura skull lacks important diagnostic characters of podocnemidids and may represent a basal pelomedusoid instead. Much of this confusion certainly originates from the highly unusual cranial and shell morphology of *Jainemys pisdurensis* as reconstructed by Jain (1977, 1986). Among others, the cranium of this taxon was originally reported to have enlarged palatines that contribute to a broad palate, pterygoids that almost completely separate the palatines from one another, an elongate, narrow basisphenoid, and opisthotics that separate the quadrates from the basisphenoid and basioccipital (Jain, 1977). These are highly unusual features that do not particularly support grouping with any other pleurodire. The shell was similarly reported to have a vase-shaped vertebral I that contributes to the anterior margin of the carapace but a nuchal that is retracted from the anterior margin of the carapace (Jain, 1986), features that are highly unusual among pleurodires as well. The possible presence of a representative of the *Shweboemys* lineage in the Late Cretaceous of India, however, has important implication regarding the temporal and spatial evolution of that group (Cadena, 2011; Pérez-García, Ortega & Murelaga, 2012; Georgalis et al., 2013; Weems & Knight, 2013).

The purpose of this contribution is to re-describe and re-evaluate all specimens that were previously referred to *Jainemys pisdurensis* comb. nov. by Jain (1977, 1986). In contrast to previous studies, we conclude that this taxon is a valid species of bothremydid turtle, which expands the known diversity of kurmademydines in the Late Cretaceous of India to three. The shell material from the Lameta Formation is diagnostic for this group as well, but cannot be assigned to a particular species. The realization that all known turtles from the Late Cretaceous of India are each other’s closest relative supports the notion that India possessed a partially endemic turtle fauna during the second part of the Cretaceous.

## Geological Settings

**Figure 1:**
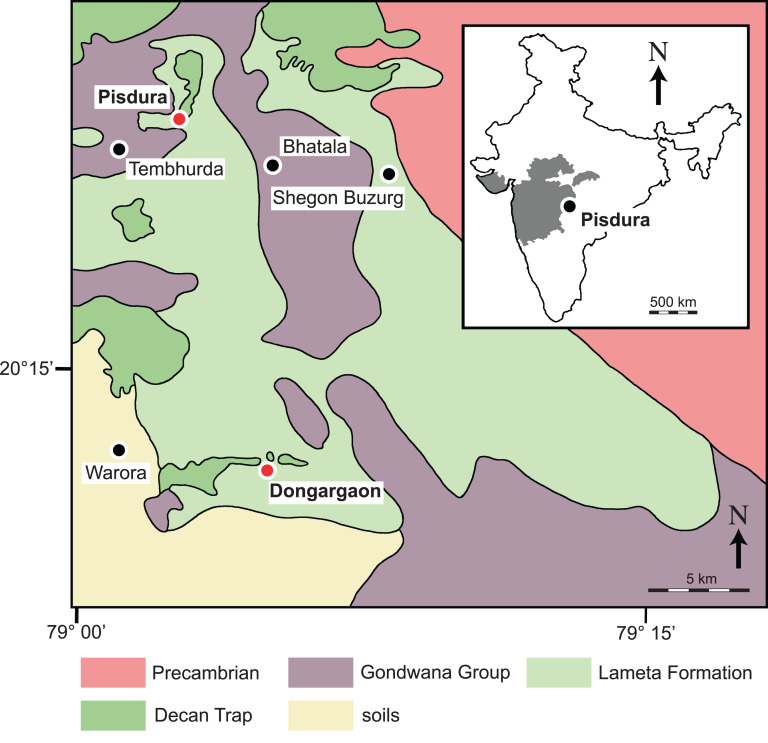
Detailed geological map of outcrops of the Lameta Formation south of Nagpur, Maharashtra, India (modified after Mohabey, Udhoji & Verma, 1993 and Khosla et al., 2016). The inset highlights the location of the map in India. Red circles denote localities that yielded specimens of *Jainemys pisdurensis* comb. nov., in particular the type locality near Pisdura village to the top left and the locality near Dongargaon village to the bottom left.

The Lameta Formation occurs in the central and western parts of India, namely in Jabalpur district in Madhya Pradesh, Nagpur and Chandrapur districts in Maharashtra, and Anjar and Kheda districts in Gujarat, covering an area of about 5,000 km^2^ (Tandon et al., 1990, 1995; Mohabey, 1996; Fernández & Khosla, 2015; Khosla & Verma, 2015). Additional, scattered outcrops of the Lameta Formation are also known from Amravati district in Maharashtra and Sagar, Amrakanthak, and Betul districts in Madhya Pradesh (Mohabey, 1996). The skull of *Jainemys pisdurensis* comb. nov. was recovered from Pisdura village (Jain, 1977) while the shells referred to this turtle were found in Dongargaon village (Jain, 1986). These two localities are located in Chandrapur District of Maharashtra, central India (Fig. 1).

The Lameta Formation of Pisdura and Dongargaon unconformably overlies the Gondwana rocks of the Satpura Basin and is overlain by the Deccan Traps (Mohabey, Udhoji & Verma, 1993). The succession in Pisdura is about 10 m thick and composed of sandstone, purple-green laminated shales, channel sandstone showing planar cross-beds, and red and green silty non-laminated sandy marls beds, all of which are characteristic of overbank deposits (Khosla et al., 2016; Kapur & Khosla, 2019). The sandstone unit at Pisdura so far produced fish teeth referable to *Igdabatis indicus*, *Enchodus* sp., and *Arius* sp. (Jain & Sahni, 1983; Prasad & Cappetta, 1993; Mohabey, 1996), the skull of *Jainemys pisdurensis* (Jain, 1977), vertebrae of the snake *Madtsoia pisdurensis* (Mohabey, Head & Wilson, 2011), and dinosaur eggshell fragments of the ootaxon *Fusioolithus baghensis* (Khosla & Sahni, 1995; Fernández & Khosla, 2015). Skeletal elements of a non-avian dinosaur, *Laplatosaurus madagascariensis*, were described by von Huene & Matley (1933), but Wilson & Upchurch (2003) more recently concluded this to be a non-valid species of titanosaur. The Lameta sediments of Pisdura furthermore yielded coprolites, bivalves, gastropods, and ostracods (Khosla et al., 2015).

In Dongargaon, the Lameta Formation is about 12 m thick and consists of sandy marls followed upward by sandy clays and shales (Kapur & Khosla, 2019). The invertebrate fauna consists of ostracods (Khosla et al., 2015, 2016) and mollusks (Mohabey, Udhoji & Verma, 1993; Mohabey & Udhoji, 1996), while the fish fauna consists of *Lepidotes deccanensis* (Sykes, 1851), *Lepisosteus indicus*, *Pycnodus lametai*, *Eoserranus hislopi* (Woodward, 1908), *Igdabatis indicus* (Prasad & Cappetta, 1993), *Cluppea* sp. (Mohabey, 1996), and *Enchodus* sp. (Jain & Sahni, 1983; Mohabey, 1996). The tetrapod fauna recovered to date includes an unidentified crocodile (Kapur & Khosla, 2019) and snake (Kapur & Khosla, 2019), the turtle shell material discussed herein (Jain, 1986), the sauropod dinosaurs *Isisaurus colberti* and Titanosauriformes indet (Jain & Bandyopadhyay, 1997; Wilson & Upchurch, 2003), and unidentified egg shell fragments and coprolites (Jain, 1989; Khosla et al., 2015; Khosla et al., 2016).

The Lameta successions are considered to be fluvial-lacustrine deposits (von Huene & Matley, 1933; Brookfield & Sahni, 1987). However, a possible marine origin on the basis of presence of algal-like structures, thalassinoid burrows, and glauconitic beds in Lameta sediments exposed near Jabalpur has been suggested by some workers (Chanda, 1963a, 1963b, 1965, 1967; Chanda & Bhattacharya, 1966; Singh, 1981; Singh & Srivastava, 1981). Brookfield & Sahni (1987) later discarded this view and concluded that the Lameta sediments were deposited in an alluvial plain environment under semi-arid conditions, which has since been corroborated by Tandon et al. (1990), Mohabey, Udhoji & Verma (1993), Mohabey & Udhoji (2000), and Mohabey & Samant (2003), among others. The rich terrestrial flora and fauna recovered from the Lameta Formation, including rich skeletal remains of sauropod and theropod dinosaurs as well as their eggs, nests, and coprolites, similarly indicate a semi-arid climate and fluvial-lacustrine environments (Mohabey, 1987; Mohabey, 1996; Srivastava & Mankar, 2015; Tandon et al., 1995). A Maastrichtian age has been suggested for the Lameta Formation of Pisdura and Dongargaon on the basis of dinosaur skeletal remains, nests, eggs, coprolites, ostracods, charophytes, diatoms, and plants (Mohabey, Udhoji & Verma, 1993; Ambwani et al., 2003; Khosla et al., 2015).

## Materials and Methods

### Materials

This study is based on all turtle specimens that had previously been described by Jain (1977, 1986) from the Lameta Formation of Maharashtra State, India, in particular ISI R200, the holotype of *Jainemys pisdurensis* comb. nov. (Jain, 1977), and ISI R185–R193, specimens initially referred to *Shweboemys pisdurensis* (Jain, 1986). The holotype of *Jainemys pisdurensis*, a skull, was collected near the village of Pisdura in Maharashtra State. All remaining specimens, all postcranial, were collected from the nearby village of Dongargaon (see “Geological Settings”).

**Figure 2:**
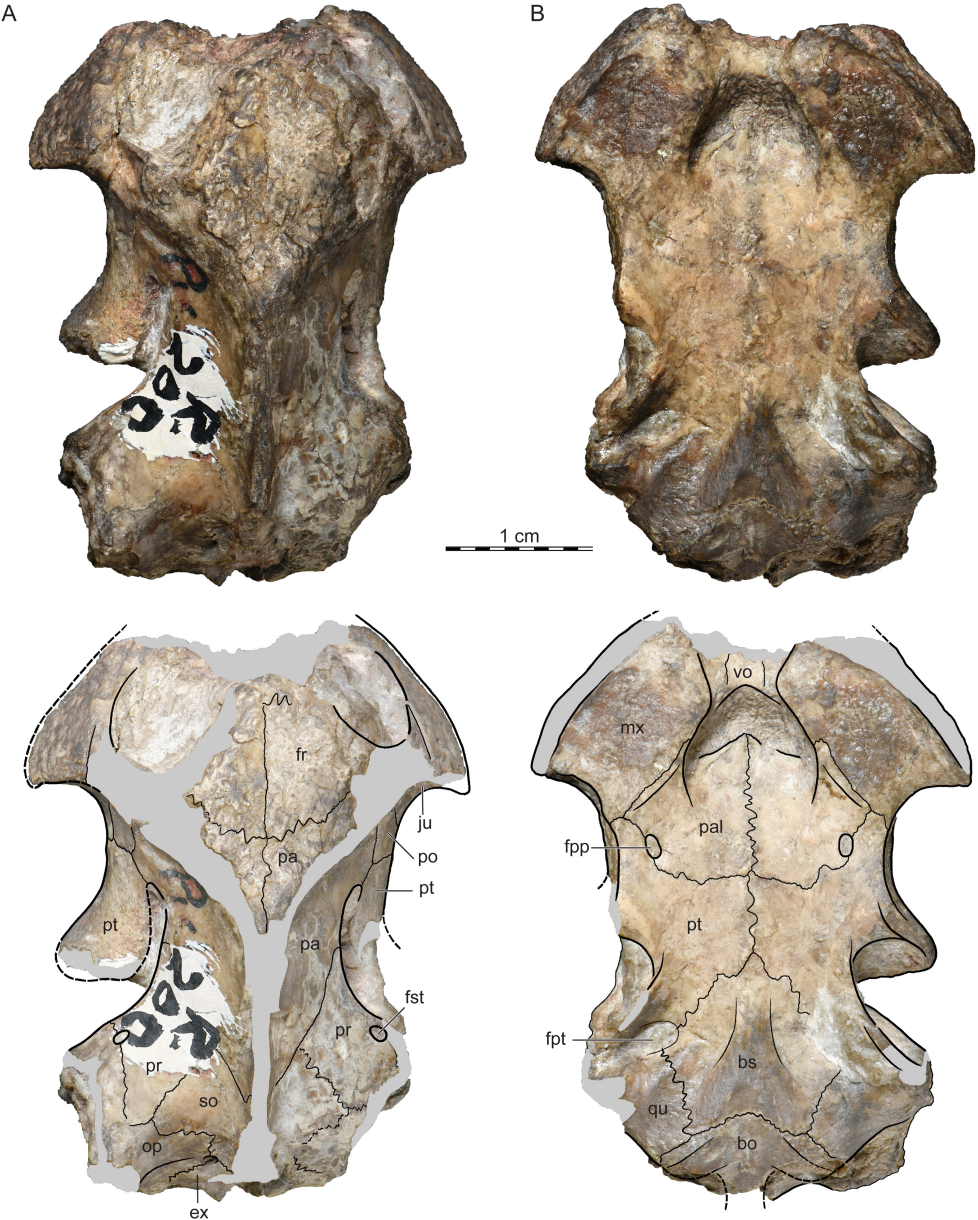
ISI R200, *Jainemys pisdurensis* comb. nov., holotype, Maharashtra, India, Lameta Formation, Late Cretaceous (Maastrichtian). Photographs and illustrations of eroded cranium in (A) dorsal and (B) ventral view. Abbreviations: *bo*, basioccipital; *bs*, basisphenoid; *ex*, exoccipital; *fpp*, foramen palatinum posterius; *fr*, frontal; *fst*, foramen stapedio-temporale; *ju*, jugal; *mx*, maxilla; *op*, opisthotic; *pa*, parietal; *po*, postorbital; *pr*, prootic; *pt*, pterygoid; *qu*, quadrate; *so*, supraoccipital; *vo*, vomer.

ISI R200: The holotype of *Jainemys pisdurensis* comb. nov. consists of a cranium (Fig. 2) that was collected near the village of Pisdura. Although the specimen was likely buried intact, most of its margins were eroded, likely prior to collecting. While much of the braincase and palate are preserved intact, the external nares, the labial ridges, most of the skull roof, and the middle ear are missing.

**Figure 3:**
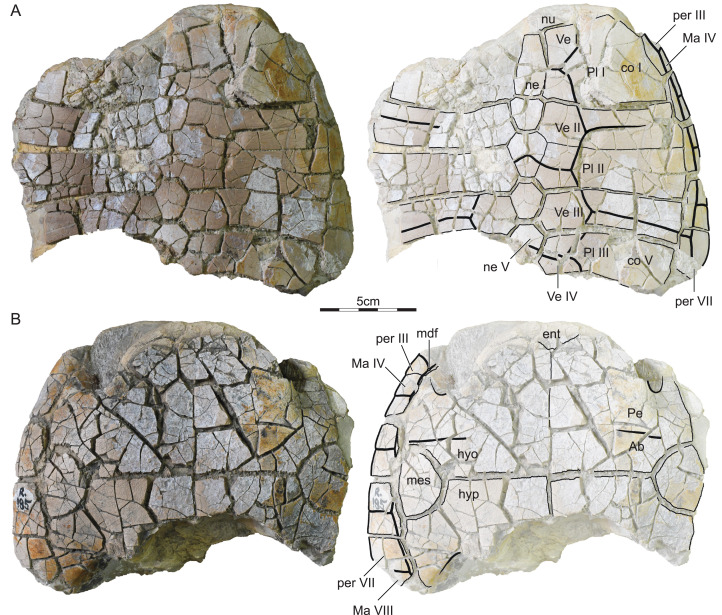
ISI R185, Kurmademydini indet., Maharashtra, India, Lameta Formation, Late Cretaceous (Maastrichtian). Photographs and illustrations of partial shell in (A) dorsal and (B) ventral view. Abbreviations: *Ab*, abdominal scute; *co*, costal; *hyo*, hyoplastron; *hyp*, hypoplastron; *Ma*, marginal scute; *mdf*, musk duct foramen; *mes*, mesoplastron; *ne*, neural; *per*, peripheral; *Pl*, pleural scute; *Ve*, vertebral scute.

ISI R185: This specimen from Dongargaon is a partial shell that includes part of the nuchal, neurals I–V, left costals I–IV, right costals I–V, peripherals III–VII, part of the entoplastron, the mesoplastra, and most of the hyo- and hypoplastra (Fig. 3). The shell shows much cracking, which was likely caused by the swelling of clays during weathering. All sutures are clearly visible, but surficial weathering of the plastron and carapace obscures some sulci.

**Figure 4:**
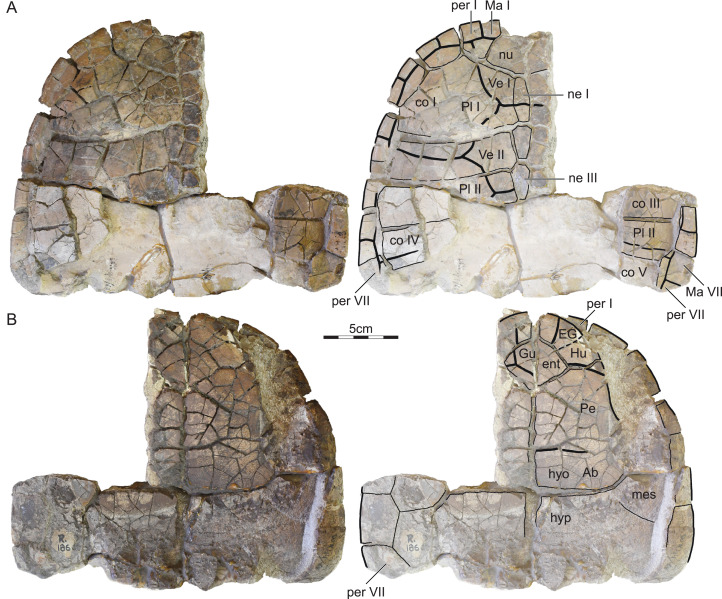
ISI R186, Kurmademydini indet., Maharashtra, India, Lameta Formation, Late Cretaceous (Maastrichtian). Photographs and illustrations of partial shell in (A) dorsal and (B) ventral view. Abbreviations: *Ab*, abdominal scute; *co*, costal; *EG*, extragular scute; *ent*, entoplastron; *Gu*, gular scute; *Hu*, humeral scute; *hyo*, hyoplastron; *hyp*, hyoplastron; *Ma*, marginal scute; *mes*, mesoplastron; *ne*, neural; *nu*, nuchal; *Pe*, pectoral scute; *per*, peripheral; *Pl*, pleural scute; *Ve*, vertebral scute.

ISI R186: This partial shell from Dongargaon consists of most of the nuchal, neurals I–III, most of left costals I–IV, the distal portions of right costals III–V, left peripherals I–VII, right peripherals VI–VII, the entoplastron, parts of the right epi-, hyo-, meso-, and hypoplastron, most of the left epi- and hyoplastron, and parts of the left meso- and hypoplastron (Fig. 4). This specimen, too, shows extensive cracking, that was likely caused by the swelling of clays during weathering. While sulci are clearly apparent on the dorsal side of the specimen, most are obscured by surficial weathering on the ventral side.

**Figure 5:**
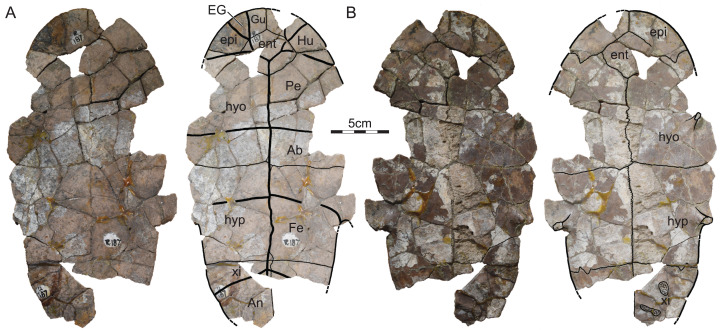
ISI R187, Kurmademydini indet., Maharashtra, India, Lameta Formation, Late Cretaceous (Maastrichtian). Photographs and illustrations of partial plastron in (A) ventral and (B) dorsal view. Abbreviations: *****Ab*, abdominal scute; An, anal scute; *EG*, extragular scute; *ent*, entoplastron; epi, epiplastron; Fe, femoral scute; *Gu*, gular scute; *Hu*, humeral scute; *hyo*, hyoplastron; *hyp*, hyoplastron; *Pe*, pectoral scute; xi, xiphiplastron.

ISI R187: This partial plastron from Dongargaon can be observed both in ventral and in dorsal view (Fig. 5). It consists of most of the median portions of the epi-, ento-, hyo-, hypo-, and xiphiplastra. The bridge is missing completely. Although the surface is slightly damaged, all sutures and sulci are clearly visible.

**Figure 6:**
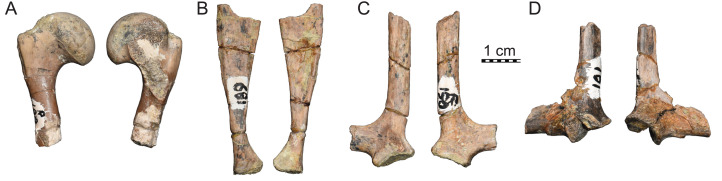
ISI R187, Kurmademydini indet., Maharashtra, India, Lameta Formation, Late Cretaceous (Maastrichtian). Isolated girdle and limb bones: ****(A) a humerus (ISI R188); (B) a partial coracoid (ISI R189); (C) a partial scapula (ISI R190); (D) a partial scapula (ISI R191).

ISI R188–R193: These six catalog numbers refer to isolated elements from Dongargaon, in particular the proximal portions of a humerus (ISI R188, Fig. 6A), a partial coracoid (ISI R189, Fig. 6B), two partial scapulae (ISI R190, Fig. 6C and ISI R191, Fig. 6D), and humerus with associated pectoral girdle (ISI R192, not figured). Jain (1986) also reported a partial ilium (ISI R193), but this is inconsistent in its morphology with that of a pleurodiran turtle and therefore not figured herein. All specimens show signs of damage, likely due to weathering.

### Nomenclatural acts

The electronic version of this article in Portable Document Format (PDF) will represent a published work according to the International Commission on Zoological Nomenclature (ICZN), and hence the new names contained in the electronic version are effectively published under that Code from the electronic edition alone. This published work and the nomenclatural acts it contains have been registered in ZooBank, the online registration system for the ICZN. The ZooBank Life Science Identifiers (LSIDs) can be resolved and the associated information viewed through any standard web browser by appending the LSID to the prefix http://zoobank.org/. The LSID for this publication is: urn:lsid:zoobank.org:pub:4842CB09-49CA-4C53-A298-32568EDE1FFF and that of the new genus is: urn:lsid:zoobank.org:act:7C288063-2F1A-4A45-BCD6-5EB95CFAEC8E. The online version of this work is archived and available from the following digital repositories: PeerJ, PubMed Central and CLOCKSS.

## Systematic Paleontology

TESTUDINES Klein, 1760

PLEURODIRA Cope, 1865

PELOMEDUSOIDES de Broin, 1988

BOTHREMYDIDAE Baur, 1891

KURMADEMYDINI Gaffney, Tong & Meylan, 2006

***Kurmademydini* indet.**

Referred material.—ISI R185–R193 (Figs. 3–6), postcranial material referred to *Shweboemys pisdurensis* by Jain (1986).

Comments.—see Discussion (below) for rationale for referring this material to Kurmademydini indet.

***Jainemys* gen.** **nov.**

Type species.—*Carteremys pisdurensis* Jain, 1977.

Diagnosis.—As for type and only species (see below).

Etymology.—In honor of Sohan Lall Jain, former professor at the Indian Statistical Institute, who collected and described the type specimen of *Jainemys pisdurensis*.

***Jainemys pisdurensis* comb.** **nov. (Jain, 1977)**

*Carteremys pisdurensis* Jain, 1977 - type description

*Shweboemys pisdurensis* Jain, 1986 - new combination

Holotype.—ISI R200, a partial cranium (Jain, 1977, figs. 2–6, pl. 1; Fig. 2).

Type locality and horizon.—Near Pisdura village, south of Nagpur, Maharashtra State, India (Fig. 1); Lameta Formation, Late Cretaceous (Maastrichtian).

Referred material.—none.

Emended diagnosis.—*Jainemys pisdurensis* comb. nov. can be diagnosed as a representative of Bothremydidae by the combination of the following characters: wide prefrontals (as opposed to Pelomedusidae and Euraxemydidae); expanded triturating surfaces with moderate palatine contribution; absence of a cavum pterygoidei (as opposed to Podocnemididae); likely presence of a small ventral exposure of the prootic at the junction between basisphenoid, pterygoid, and quadrate; and basisphenoid-quadrate contact (also in Podocnemididae) and as a representative of Kurmademydini by the likely presence of extremely deep upper temporal emargination, jugal contribution to orbit (as opposed to Cearachelyini), pentagonal basisphenoid (as opposed to Cearachelyini), and the likely placement of mandibular condyle anterior to basioccipital (diagnostic character taken from Gaffney, Tong & Meylan, 2006). *Jainemys pisdurensis* furthermore differs from podocnemidids by lacking an interorbital groove and an interparietal scale. Among kurmademydines, *Jainemys pisdurensis* can be differentiated from *Kurmademys kallamedensis* by having a more elongate skull, a greater posterior extension of the frontals, more anteroposteriorly elongate palatines that only contribute minorly to the triturating surfaces, remnants of the vomer between the maxillae, presence of a prootic and opisthotic contact in the upper temporal fossa, which hinders contact between the quadrate and supraoccipital, a more equilaterally pentagonal basisphenoid, and a much smaller fossa pterygoidea and from *Sankuchemys sethnai* by having a more elongate skull, a greater posterior extension of the frontals, presence of a prootic and opisthotic contact in the upper temporal fossa, which hinders a contact between the quadrate and supraoccipital, broader triturating surfaces, by lacking a posterior vomeral process that contacts the palatines, and by having more expanded palatines, a more equally sided pentagonal basisphenoid, and a shorter basioccipital.

## Results

### Description of cranial material from the Lameta Formation

#### Outline and proportions

The holotype of *Jainemys pisdurensis* comb. nov., ISI R200, is badly damaged and many structures of interest cannot be assessed, most notably the shape of the external nares and the middle ear region (Fig. 2). Even though the skull looks to be deeply emarginated, the near-complete weathering of the temporal bones precludes assessing the presence or depth of either the lower or upper temporal emarginations, although the thin nature of the bone at the breaks is suggestive of a deep upper temporal emargination. However, by comparison to other pelomedusoids, we conclude that the skull was relatively flat, longer than broad, that the orbits were mostly oriented dorsally, that the interorbital space is broad, and that the triturating surfaces were expanded and flat.

#### Nasals

The dorsal margin of the external nares of ISI R200 is damaged (Fig. 2A). We are therefore not able to comment on the presence and morphology of this bone. The phylogenetic placement of *Jainemys pisdurensis* within Pelomedusoides (see “Phylogenetic Analysis”), however, predicts that this taxon lacked nasals (Gaffney, Tong & Meylan, 2006).

#### Prefrontals

Only the most posterior portions of the prefrontals are preserved on the dorsal skull roof, just anterior to the midline contact of the frontals (Fig. 2A). Jain (1977) indicated the presence of larger prefrontals in the interorbital space, but we interpret his prefrontal/frontal suture as damage to the specimen. The descending branch of the prefrontal is likely preserved but obscured by the matrix that fills the orbits. As little is known about the prefrontals, the relatively wide interorbital space suggests that they were wide elements. Also, an interorbital groove is clearly absent.

#### Frontals

Much of the frontals is preserved in ISI R200, but it is difficult to discern their sutures with confidence (Fig. 2A). A suture is developed at the anterior margin of the right frontal that likely represents its sutures with the prefrontal. An oblique suture at the posterior margin of both frontals suggests a broad, contact with the parietals. Jain (1977) reported that the frontals broadly contact the postorbitals laterally, but we find the relevant area to be too damaged to allow confirming or refuting this observation. As the anterior suture of the frontal with the prefrontal and the lateral suture of the frontal with the postorbital cannot be ascertained with confidence, it is not clear, if the frontal contributes to the margin of the orbit, as suggested by Jain (1977), although the geometry of the skull makes this rather likely. There is no evidence in the form of a sulcus for the presence of an interparietal scale.

#### Parietals

Small remnants of the dorsal plate of the parietals are preserved just posterior to the frontals (Fig. 2A). In contrast to Jain (1977), we conclude that the lateral and posterior margins of the dorsal plate of the parietal are damaged. We are therefore not able to ascertain the likely presence of a lateral contact of the parietal with the postorbital. The broad descending process of the parietal contacts the postorbital and pterygoid at the base of the processus trochlearis pterygoidei (Fig. 2A) and the prootic and supraoccipital within the upper temporal fossa. A likely contribution to the trigeminal foramen cannot be ascertained, as it is covered by matrix. The contacts of the descending process broadly agree with Jain’s (1977) original observations.

#### Jugal

The dorsal surface of both jugals are damaged (Fig. 2). The anterior process nevertheless clearly contributes to the posterior margin of the orbit, while the lateral process frames the anterior margin of the lower temporal fossa, just below the posterior margin of the maxilla, and contacts the postorbital medially and the pterygoid posteriorly at the base of the processus trochlearis pterygoidei. Though plausible, we are not able to confirm the broad, dorsal contact of the jugal with the postorbital on the dorsal skull surface, as indicated by Jain (1977).

#### Quadratojugal

The quadratojugals were eroded completely (Fig. 2).

#### Squamosal

The squamosals are not preserved (Fig. 2). The part of the skull identified by Jain (1977) as the squamosal in fact represents the opisthotic.

#### Postorbital

A portion of the dorsal plate of the postorbital must be present posterior to the orbits, but the bone is too damaged to allow assessing its contacts or posterior extent (Fig. 2). As in all pleurodires, the postorbital forms a ventral process that forms the posterior wall of the orbit. In ISI R200 the process contacts the parietal, pterygoid, and jugal anterior to processus trochlearis pterygoidei. Much of this ventral process was interpreted as the jugal by Jain (1977).

#### Premaxilla

The premaxillae are missing completely (Fig. 2).

#### Maxilla

In dorsal view, the maxilla forms the lower margin of the orbit and contacts the jugal posteriorly (Fig. 2). Its likely anterior contacts with the prefrontal and premaxilla are not preserved. In ventral view, the maxillae contact the vomer anteromedially, the palatines posteromedially, the pterygoids posteriorly, and form notably broad, but flat triturating surfaces that lack a midline contact with one another. Clear damage to the lateral margins of both maxillae suggests that a distinct labial margin was present that laterally framed the flat portions of the palate. The narrowness of the damage indicates, however, that the labial margin was relatively low and that the maxilla was not higher than the orbit in lateral view. There are no traces of lingual ridges. The broadly triturating surfaces together frame a broad, medial tongue grove that expands towards the anterior. Our observations differ from those of Jain (1977), but suggesting that the palatine only form a minor contribution to the posteromedial margins of the triturating surfaces.

#### Vomer

A portion of the vomer is preserved in ventral view between the triturating surfaces of the maxillae (Fig. 2B). Likely anterior contacts with the premaxillae are not preserved. As the posterior process of the vomer is lacking, the internal choanae are fully confluent with one another. In contrast to Jain (1977), we neither find a posterior contact of the vomer with the palatines, nor with the pterygoids.

#### Palatine

The palatines are large, subrectangular elements that broadly roof the palate (Fig. 2B). The anterior margins of the palatines taper to form a midline process between the triturating surfaces. A contact with the vomer is absent. The palatine anterolaterally contacts the maxilla and narrowly contributes to the posteromedial margin of the flat triturating surface. The palatine finally contacts the pterygoid along a broad posterolateral and a broad posterior suture. The foramen palatinum posterius is located in the suture between the palatine and the pterygoid. Our interpretation of the palatine differs substantially from that of Jain (1977), by recognizing a broad midline contact, a greater extent to the posterior, but a smaller extent to the anterior, no contact with the vomer, and only a minor contribution to the triturating surfaces.

#### Quadrate

Only the medial portions of both quadrates are preserved (Fig. 2). We, therefore, cannot comment on the morphology of the cavum tympani, incisura columella auris, and condylus articularis. In dorsal view, the medial portion of the quadrate broadly contacts the prootic to form the foramen stapedio-temporale, which is located near the anterior margin of the otic capsule. Although the remaining contacts are unclear within the upper temporal fossa, a clear contact between the opisthotic and prootic precludes contact of the quadrate with the supraoccipital. In ventral view, the quadrate broadly floors the cavum acustico-jugulare. The quadrate broadly contacts the basisphenoid anteromedially and the basioccipital posteromedially. We are not able to clarify if a possible contact is present with the exoccipital posteriorly or with the prootic and pterygoid anteriorly. A pit filled with matrix on both sides of the skull suggests the presence of a small, but distinct fossa pterygoidea (sensu Gaffney, Tong & Meylan, 2006). The likely entry path of the carotid and facial nerve systems is covered by matrix. The mandibular condyle is missing on both sides of the skull, but the intact posterior buttress of this region on the left side of the skull strongly suggests that the condyles were located anterior to the basioccipital.

#### Pterygoid

The pterygoids are generally well preserved (Fig. 2). In ventral view, the pterygoid broadly contacts the palatine anteriorly along a straight suture, anteromedially along an oblique suture, and contributes to the lateral margins of the foramen palatinum posterius. The anterior process of the pterygoid furthermore has a short contact with the maxilla. The gliding surface of the robust processus trochlearis pterygoidei is oriented at an angle of 45 degrees relative to the midline of the skull. In dorsal view, anterior contacts are apparent at the base of the process trochlearis pterygoidei with the jugal, postorbital, and parietal. The pterygoids have a broad posteromedial contact with the basisphenoid, but possible posterior contacts with the prootic and quadrate are obscured. The posteromedial aspects of the pterygoid are damaged, but the presence of a low pterygoid flange (sensu Gaffney, Tong & Meylan, 2006) can be ascertained. Our interpretation differs substantially from than of Jain (1977), but suggesting that the pterygoids do not broadly separate the palatines from one another.

#### Supraoccipital

What remains of the supraoccipital is best observed in dorsal view (Fig. 2). It here forms a broad anterolateral process that roofs much of the otic capsule and contacts the parietal anteromedially, the prootic anterolaterally, the opisthotic posterolaterally, and the exoccipital posteriorly. We are not able to assess the shape and extent of the crista supraoccipitalis and the margins of the foramen magnum due to damage. Our observations mostly differ from those of Jain (1977) by suggesting that the supraoccipital does not have a broad anterior extension along the midline.

#### Exoccipital

The exoccipitals are badly damaged and we, therefore, can only poorly assess their morphology (Fig. 2). A clear anterolateral and anteromedial contact is apparent with the opisthotic and supraoccipital, respectively in dorsal view. The exoccipital likely contributes to the margin of the foramen magnum, but it is unclear if a contact exists with the quadrate. The occipital condyle has eroded fully.

#### Basioccipital

The basioccipital is about three times wider than long (Fig. 2B). It anteriorly has a convex contact with the basisphenoid, and short anterolateral contacts with the quadrate. The dorsal contacts with the exoccipitals are obscured. The basioccipital tubercles are damaged on both sides of the skull. The ventral surface of the bone is only ornamented by a subtle, median depression.

#### Prootic

The prootics are well preserved, but matrix obscured their ventral aspects (Fig. 2). The size of the covered area, however, suggests that its ventral exposure was likely minor. In dorsal view, the prootic broadly contacts the parietal anteromedially, the supraoccipital posteromedially, the quadrate laterally, and the opisthotic posteriorly. The foramen stapedio-temporale is jointly formed by the prootic with the quadrate and is situated near the anterior margin of the otic capsule. These observations broadly agree with those of Jain (1977), with the exception of the broad lateral contact with the quadrate.

#### Opisthotic

Only the medial portions of the opisthotic can be observed in dorsal view (Fig. 2). It here contacts the exoccipital posteriorly, the supraoccipital anteromedially, the prootic anteriorly, and, likely, the quadrate anterolaterally. Jain (1977) indicated that the opisthotic contacts the pterygoid, quadrate, and basioccipital on the ventral side of the skull, but we interpret this portion of the skull as the quadrate only.

#### Basisphenoid

The basisphenoid is a large, pentagonal element with this slightly longer than wide (Fig. 2B). It has equally sided anterolateral contacts with the pterygoids, posterolateral contacts with the quadrate, and a convex posterior contact with the basioccipital. Likely lateral contacts with the prootics within the fossa pterygoidea are obscured by matrix. Our interpretation of the basisphenoid from that of Jain (1977), who saw a narrow element with broad lateral contacts with the pterygoids only.

### Description of postcranial material from the Lameta Formation

#### Outline, proportions, and texture

The three available shell specimens are incomplete obscuring most details pertaining to the outline and proportions (Figs. 3–5). We nevertheless conclude that the shells probably had a rounded outline, that a deep nuchal notch is absent, and that the plastral lobes have similar dimensions. From what is preserved, it appears that the anterior plastron lobe protrudes beyond the anterior margins of the carapace in ISI R185, but only reaches the anterior margin of the shell in ISI R186. These differences may have taxonomic significance, or be the result of differential crushing. All shells are externally decorated by the finely vermiculated texture that is characteristic of most pleurodires.

#### Nuchal

Only the posterior portion of the nuchal is preserved in ISI R185 and ISI R186 (Figs. 3 and 4). What remains documents that the nuchal has a broad anterolateral contact with peripheral I, a broad posterolateral contact with costal I, and short, posterior contact with neural I. The surrounding elements indicate that a broad nuchal notch was absent, but it remains unclear if the nuchal itself framed a small nuchal notch. Assuming that the anterior margin of peripheral I corresponds to the anterior margin of the nuchal, we conclude that the nuchal is slightly wider than long. Jain (1986) suggested that the nuchal does not contribute to the anterior margin of the shell, a feature that would be unique among pleurodires. Comparison with other turtles suggests instead that the nuchals are damaged.

#### Neurals

ISI R185 preserves neurals I–V and ISI R186 neurals I–III (Figs. 3 and 4). Although the intact posteromedial margin of costal V indicates that costal VI was present in this specimen as well, we cannot comment on the possible presence of neurals VII and VIII. In both specimens, neural I is a rectangular element with slightly convex lateral margins that is narrower than all subsequent elements, at least as preserved, but about 50% longer. Neural I is notably broader in ISI R185 than in ISI R186. The remaining, preserved neurals are hexagonal with shorter anterolateral than posterolateral sides. In ISI R185, the neurals are narrow and have shorter anterolateral sides, than in ISI R186. Jain (1986) reported that neural I is nearly hexagonal and about as broad as the posterior neurals, but the available material clearly contradicts this interpretation.

#### Costals

ISI R185 and ISI R186 preserves most of costals I–V (Figs. 3 and 4). Costal I is a particularly anteroposteriorly expanded element that is about as mediolaterally wide but about twice as anteroposteriorly long as the sequent costal elements. It contacts the nuchal anteromedially, peripherals I–IV anteriorly and laterally, costal II posteriorly, and neural I medially. A likely ventral contact with the axillary buttress is blocked from view. Costals II–V are elongate, rectangular elements that medially contact two neurals each. In particular, costal II laterally contacts peripherals IV and V, costal III contacts peripherals V and VI, costal IV contacts peripherals VI and VII, and costal V contacts peripherals VII and VIII. The likely ventral contact of the costal series with the inguinal buttress is obscured by matrix as well.

#### Peripherals

ISI R185 includes most of right peripherals III–VII and ISI R186 most of left peripherals I–VII and right peripherals VI and VII (Figs. 3 and 4). The contacts with the nuchal and costals are described above, the contacts with the plastron below. Although peripheral VIII is missing, ISI R185 reveals that the bridge spanned from peripheral III to peripheral VIII. At least one musk duct foramen is apparent between peripheral III and the axillary buttress at the junction of marginals III and IV and the skin sulcus.

#### Suprapygals and Pygal

No pygal elements are preserved.

#### Carapacial Scutes

ISI R185 and ISI R186 jointly provide evidence for vertebrals I–IV, pleurals I–III, and marginals I–VIII (Figs. 3 and 4). The phylogenetic position of *Jainemys pisdurensis* predicts that a cervical was absent. Vertebral I is a trapezoidal element that is broader anteriorly than posteriorly. Vertebral I anteriorly contacts all of marginal I and the medial third of marginal II. Vertebrals II and III are hexagonal elements that are about as wide as long. In ISI R185, the lateral sides of these elements form a clear, obtuse angle and the two available intervertebral sulci, that is, those between vertebral II and III and vertebral III and IV, show a deep anterior inflection along the midline (Fig. 3). In ISI R186, the lateral side of vertebrals II and III taper to form distinct points, but the only available intervertebral sulcus, that between vertebral I and II, shows no anterior inflection. These differences might have taxonomic value (see “Discussion” below). While the preserved intervertebral sulci are located on neurals I, III, and V, the interpleural sulci are located on costals II and IV. Pleural I anteriorly and laterally contacts the lateral two-thirds of marginal II, all of marginals III and IV, and the anterior third of marginal V. Pleural II laterally contacts the posterior two-thirds of marginal V, all of marginal VI, and the posterior half of marginal VII. Pleural III at least contacts the posterior half of marginal VII and all of marginal VIII. The marginals are rectangular elements that evenly dissect the peripherals and that do not lap onto the costals. Jain (1986) suggested that the first vertebral contributes to the anterior margin of the shell, but he was apparently misled by damage to ISI R186.

#### Plastral bones

The plastron consists of the entoplastron and paired epi-, hyo-, meso-, hypo-, and xiphiplastral (Figs. 3–5). The epiplastra and entoplastron are best preserved in ISI R187. The entoplastron is a large, rhomboidal element with similarly sized anterolateral and posterolateral contacts with the surrounding epiplastral and hyoplastra. It is clearly wider than long. The epiplastra are large elements, each being about the same size as the entoplastron. They jointly form the rounded anterior plastral margin and form a straight posterior contact with the hyoplastron and a straight median contact with one another. The hyo- and hypoplastral jointly form the majority of the plastron. The bridge, by contrast, also includes subtriangular mesoplastra. The hyoplastron laterally contacts the posterior half of peripheral III, all of peripheral IV, and the anterior half of peripheral V. The inguinal buttress apparently contacts the costals, but the extent of this contact remains unclear. The mesoplastron laterally contacts the posterior half of peripheral V and the anterior two thirds of peripheral VI. The hyoplastron laterally contacts the posterior half of peripheral VI, all of peripheral VII, and, likely, the anterior half of peripheral VIII. Here, too, contact with the costals is evidence, but not the details of this contact. The xiphiplastra, only preserved in ISI R187 form the posterior half of the posterior plastra lobe and have a straight anterior contact with the hypoplastra. The likely presence of an anal notch cannot be confirmed, as the relevant portion of the bone is damaged in ISI R187. Two articular scars on the dorsal side of the xiphiplastra evidence a former sutural articulation with the pubis and ischium. Our observations of the plastron broadly agree with those of Jain (1986).

#### Plastral scutes

The three available specimens jointly document a single gular and paired extragulars, humerals, pectorals, abdominals, femorals, and anals (Figs. 3–5). Inframarginals are absent. The gular elements are best preserved in ISI R187 (Fig. 5). The single gular element is larger than each humeral. It broadly covers the medial thirds of the epiplastra and anterior two-thirds of the midline of the entoplastron. The gular laterally contacts the extragulars and humerals, posterolaterally contacts the pectorals, and contributes to the anterior margin of the plastron. The extragulars are small, triangular elements that do not cover the entoplastron. The extragulars contact the gular medially and the humerals posterolaterally. The humerals are unusually small, polygonal elements that form much of the anterolateral margin of the plastron and minorly cover the entoplastron. They contact the extragulars and the gular anteromedially and the pectoral posteromedially, but lack a midline contact with one another. The scute-skin sulcus of the gulars and humerals runs parallel to the margin of the anterior plastral lobe. The plastron therefore lacks a dorsal lip. The pectorals are rectangular elements that broadly cover the posterolateral portions of the entoplastron, laterally contact marginals IV and V, and form a straight posterior sulcus with the abdominals that is located just anterior to the mesoplastra. The abdominals are rectangular elements that fully cover the mesoplastra, anteriorly contact the pectorals, posteriorly contact the femorals, contribute to the axillary notch, and laterally contact the posterior tip of marginal V, all of marginals VI and VII, and the anterior portions of marginal VIII. The femorals are the most anteroposteriorly elongate elements. They contact the abdominals anteriorly and the anals posteromedially. The anals are clearly restricted to the xiphiplastra but damage obscures a possible contribution to a deep anal notch. Our observations pertaining to the plastral scute of the shell material from the Lameta Formation broadly agree with those made by Jain (1986).

#### Limbs and girdles

The associated limb bones and girdle elements are fragmentary and do not provide much useful character evidence (Fig. 6). What remains of the humerus suggests that it is a gracile element, that the head is rounded, and that the lateral process is placed close to the head (Fig. 6A). The scapulocoracoid is not fused allowing the bones to fall apart easily. The coracoid is elongate and slightly expanded distally (Fig. 6B). The scapula forms a short neck above the glenoid (Figs. 6C and 6D). No pelvis elements are preserved, but the articular scares preserved on the dorsal side of ISI R187 amply demonstrates that the pelvis was sutured to the shell (Fig. 5).

## Phylogenetic Analysis

To investigate the phylogenetic position of the turtle material from the Lameta Formation, we included it into the global pleurodire matrix of Ferreira et al. (2018), which is a concatenation and expansion of previously published matrices, in particular the chelid matrix of Bona & de la Fuente (2005), the pelomedusoid matrix of Gaffney, Tong & Meylan (2006), and the pan-podocnemidid matrix of Gaffney et al. (2011). As it is questionable whether the shell material from the Lameta Formation is referable to *Jainemys pisdurensis* comb. nov., we scored three separate terminal taxa based on the available material: (1) *Jainemys pisdurensis*, based on the skull only; (2) Lameta shells, a composite based on all available shell material from the Lameta Formation; and (3) “*Jainemys pisdurensis*,” a composite based on all turtle material from the Lameta Formation. The scoring of all coeval turtles from India and Madagascar was updated to ensure that they are scored evenly, in particular the Late Cretaceous (Maastrichtian) *Kinkonychelys rogersi* from Madagascar, as described by Gaffney, Krause & Zalmout (2009), the Late Cretaceous (Maastrichtian) *Kurmademys kallamedensis* from India, as described by Gaffney, Chatterjee & Rudra (2001) and Gaffney, Tong & Meylan (2006), the Late Cretaceous (Maastrichtian) *Sokatra antitra* from Madagascar, as described by Gaffney & Krause (2011), and the Late Cretaceous (Maastrichtian) *Sankuchemys sethnai* from India, as described by Gaffney et al. (2003). A list of changes is provided in File S1 and the final data matrix File S2. In contrast to Ferreira et al. (2018), we do not believe that character 71 forms a morphocline and therefore do not order it anymore. In contrast, characters 1, 10, 51, 52, 56, 57, 71, 75, 78, 81,82, 86, 103, 114, 115, 128, 171, 172,182, 183, 193, 195, 225, 231, and 242 form morphoclines and therefore were ordered. We furthermore were able to create additional, orderable morphoclines by swapping the character states for characters 88, 112, 130, 202, 220 and 224. The final data matrix includes 41 morphoclines than can be ordered, in particular characters 1, 10, 14, 18, 19, 51, 52, 56, 57, 75, 78, 81, 82, 86, 88, 95, 96, 99, 101, 103, 112, 114, 115, 119, 128, 129, 130, 171, 172, 174, 175, 182, 183, 193, 195, 202, 220, 224, 225, 231, 242.

**Figure 7:**
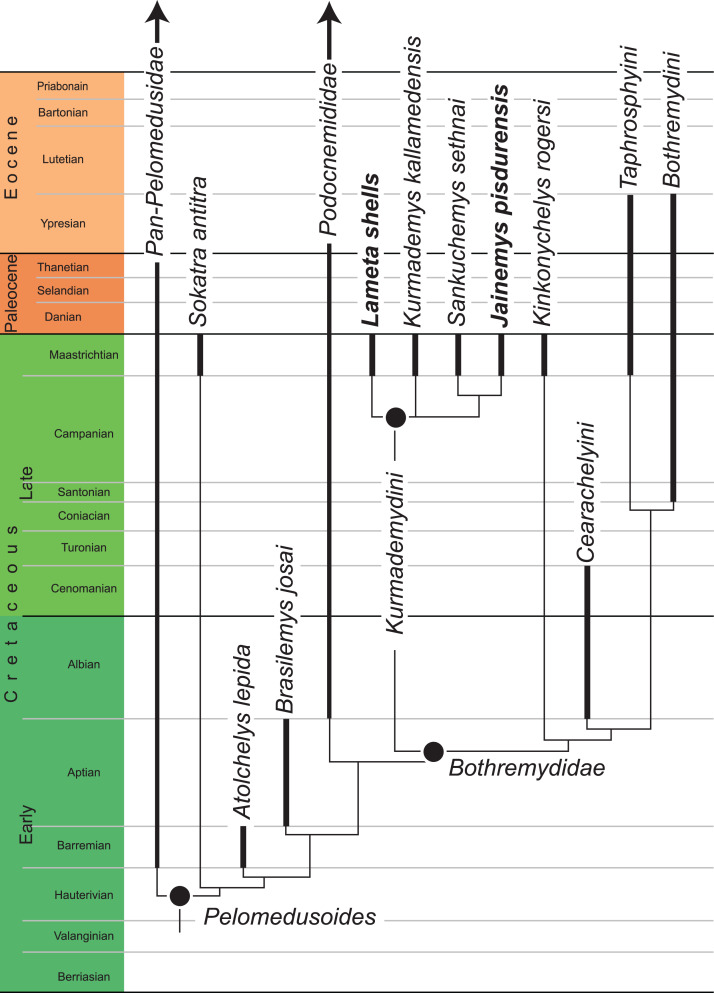
A time-calibrated summary of the strict consensus cladograms retrieved from the phylogenetic analysis. Speciose clades outside of the purview of this study are collapsed into more inclusive clades. Dark lines highlight the known temporal distribution of a species or of individuals representing clades.

Three analyses were performed that differ in their inclusion of material from the Lameta Formation (see above). In each case, the matrix was subjected to a parsimony analysis using the software TNT (Goloboff, Farris & Nixon, 2008), all morphoclines were run ordered, light implied weighting was implemented with a k value of 12 (Goloboff, Torres & Arias, 2018), and 1,000 replicates of random addition sequences were followed by a round of tree bisection and reconnection. The first analysis, which only includes the type skull of *Jainemys pisdurensis*, yielded 18 trees with a best score of 50.85348. The second analysis, which only includes the shell material from the Lameta Formation, yielded 36 trees with a best score of 50.78754. The third analysis yielded 18 trees with a best score of 50.8548. In all three cases, the “Pruned Trees” function of TNT suggested that *Cambaremys langertoni* França & Langer, 2005 from the Late Cretaceous (Maastrichtian) of Brazil acts as a regionally relevant wild card taxon. The strict consensus and 50% consensus tree of all analyses to the inclusion and exclusion of *Cambaremys langertoni* are provided in File S3. A summary of the trees retrieved from all analyses is provided in Fig. 7.

## Discussion

### Alpha taxonomy

Our revision of the cranial morphology of *Jainemys pisdurensis* comb. nov. provides an opportunity to re-evaluate the validity of named fossil turtles from the Late Cretaceous of India. All three named species were collected from sediments dated to the Maastrichtian: *Jainemys pisdurensis* (Jain, 1986) from eastern Maharashtra in the center of India, *Kurmademys kallamedensis* Gaffney, Chatterjee & Rudra, 2001 from Tamil Nadu in southeastern India, and *Sankuchemys sethnai* Gaffney et al., 2003 from western Maharashtra on the western coast of India. Although Gaffney, Chatterjee & Rudra (2001) and Gaffney et al. (2003) alluded to *J. pisdurensis*, they did not differentiate their taxa from it, as they presumed it to be a pan-podocnemidid, an assessment based on the somewhat skewed descriptions of Jain (1986), which were furnished long before the morphology of bothremydids had been studied in any detail. As our revision of this taxon, combined with our phylogenetic analysis (see below), suggests that *J. pisdurensis* is actually a kurmademydine, it is necessary to re-evaluate the validity of all taxa. We did not have the opportunity to study the relevant skull material in person, so our observations are taken instead from Gaffney, Chatterjee & Rudra (2001) and Gaffney et al. (2003). We here will not differentiate *J. pisdurensis* from coeval pan-podocnemidids, such as *Bauruemys elegans* Suárez, 1969 from the Late Cretaceous (Maastrichtian) of Brazil, as the attribution of *J. pisdurensis* to this group was never based on much data and because the differences are all too apparent (see Gaffney et al., 2011 for a thorough treatment of pan-podocnemidids).

*Kurmademys kallamedensis* is based on several skulls, of which the best preserves nearly all details in three dimensions (Gaffney, Chatterjee & Rudra, 2001). A number of differences are apparent that clearly distinguish *Jainemys pisdurensis* from *K. kallamedensis*. Although the skull of *J. pisdurensis* shows significant damage, most bones are more anteroposteriorly elongate than in *K. kallamedensis*, suggesting that the entire skull is more elongate. On the dorsal skull roof, the frontals of *J. pisdurensis* extend significantly further posteriorly. Within the upper temporal fossa, a clear contact is present on *J. pisdurensis* between the prootic and opisthotic, which hinders contact between the quadrate and supraoccipital, the opposite arrangement as seen in *K. kallamedensis*. The palate of *J. pisdurensis* resembles *K. kallamedensis* by having flat, expanded triturating surfaces, but the contribution from the palatine is much smaller, the palatines reach much deeper into the internal nares, and remnants of the vomer are present. The basicranium of *J. pisdurensis* shows a much smaller fossa pterygoidea and a more elongate, pentagonal basisphenoid, which contrasts the compressed element seen in *K. kallamedensis*.

*Sankuchemys sethnai* is only based on a single, heavily crushed skull (Gaffney et al., 2003). In overall proportions, *Jainemys pisdurensis* differs from *S. sethnai* by having more anteroposteriorly elongate bones, suggesting that the skull was more elongate. In dorsal view, the frontal of *J. pisdurensis* also appears to have a greater extension towards the posterior and the prootic contacts the opisthotic, which contrasts *S. sethnai*, which has shorter frontals and lacks a prootic-opisthotic contact, similar to *K. kallamedensis*. In ventral view, *J. pisdurensis* resembles *S. sethnai* by only having a minor contribution of the palatine to the triturating surfaces and by having a vomer, but differs by having broader triturating surfaces, lacking a posterior vomeral process that contacts the palatines, by having more expanded palatines, a more elongate, pentagonal basisphenoid, and a shorter basioccipital.

Our revision suggests that three distinct cranial morphotypes are apparent among fossil turtles from the Late Cretaceous of India. We are therefore able to confirm the validity of *Jainemys pisdurensis*, *Kurmademys kallamedensis*, and *Sankuchemys sethnai*. Jain (1977) originally referred his new turtle to *Carteremys*, which was named by Williams (1953) for the problematic fossil turtle *Testudo leithii* Carter, 1852 from the latest Cretaceous to early Paleogene (Wood, 1970) Intertrappean beds of western Maharashtra, a turtle that had already been referred by Lydekker (1890) to the chelid taxon *Hydraspis*. As presented by Williams (1953), *Carteremys leithii* has a morphology that is broadly consistent with a bothremydid in that the humerals are restricted to the anterior portion of the shell, but as all specimens have been reported lost (Jain, 1977), we agree with previous authors that this taxon should be disregarded as a nomen dubium (Gaffney, Tong & Meylan, 2006). A referral of *pisdurensis* to *Carteremys* is therefore out of the question. Jain (1986) later referred *pisdurensis* to *Shweboemys*, which is based on *Shweboemys pilgrimi* Swinton, 1939 from the Pliocene of Myanmar. As our phylogenetic analysis (see below) highlights that *pisdurensis* is a kurmademydine bothremydid, not a podocnemidid, referral to this taxon is inappropriate as well. Although it would be possible to fold all three available kurmademydine turtles from the Late Cretaceous of India into a single genus, *Kurmademys*, we here create a new genus name, *Jainemys*, as this maintains the names created by Gaffney, Chatterjee & Rudra (2001) and Gaffney et al. (2003). The new genus name is formed in honor of Prof. Sohan Lall Jain, who collected and described all specimens discussed herein.

#### Turtle shells from the Lameta Formation

Jain (1977) originally based *Jainemys pisdurensis* comb. nov. on a partial skull that had been collected from the Lameta Formation near the village of Pisdura, Maharashtra State, India. About a decade later, Jain (1986) described shell material from the Lameta Formation of the nearby village of Dongargaon, Maharashtra State, India. Although Jain (1986) noted highlighted difficulties with associating shells that had been found separately from skulls, he referred the new shell material to *Jainemys pisdurensis* without explicit justification. As no other turtles had been names from the Late Cretaceous of India, we speculate that temporal and spatial proximity played a role.

Our revision of turtle skulls from the Late Cretaceous of India (see “Alpha Taxonomy”) suggests that three distinct kurmademydine turtles are present in roughly coeval (Maastrichtian) deposits scattered across the southern half of India. Given that two out of three recognized species are only known from a single specimen (i.e., *Jainemys pisdurensis* and *Sankuchemys sethnai*) and given that extant turtles are known to occur across much greater ranges (Turtle Taxonomy Working Group (TTWG) et al., 2017), it is reasonable to speculate that these three turtles may have overlapped in their range. Indeed, given that three distinct localities have yielded three distinct turtles, it is also reasonable to expect that much more diversity is to be found. We, therefore, see no reason to presume a priori that the shells described by Jain (1986) belong to *Jainemys pisdurensis*, just because they were found in the same formation.

The poorly preserved shell material referred to *Kurmademys kallamedensis* was collected from the same quarry as the available skull material, including the type (Gaffney, Chatterjee & Rudra, 2001; Gaffney, Tong & Meylan, 2006). Although no specimens were found in articulation, we find this association to be highly reasonable. From what can be gleaned, the shell material of *K. kallamedensis* broadly agrees in its morphology with the shells from the Lameta Formation in that they are rounded, have a square neural I, hexagonal neurals II–V with short anterior sides, humerals that are restricted to the anterior portions of the anterior plastral lobe, and pectorals that do not cover the mesoplastra. A number of differences are nevertheless apparent, also within the material from the Lameta Formation. For instance, neural I of *K. kallamedensis* resembles that of ISI R185 by being relatively short, but differs from that of ISI R186, which is notably long. Neurals II–V of *K. kallamedensis*, on the other hand, resemble ISI R186 by being relatively narrow, but differ from those of ISI R185, which are notably broad and blocky. Additional differences may also be apparent in the shape of the vertebrals, but comparisons are difficult for the moment, given the idealized description of the *K. kallamedensis* shell material. As the amount of interspecific variation is unclear in kurmademydine turtles, we are only left to conclude that up to three shell morphotypes are apparent, two of which from the Lameta Formation, for which we can only refer one to *K. kallamedensis* based on geographic provenience. Future finds will hopefully clarify if any of the shell material described by Jain (1986) is indeed referable to *Jainemys pisdurensis*. Until then, we diagnose all shell material from the Lameta Formation as Kurmademydini indet.

#### Phylogenetic relationships, biogeography, and paleoecology

We performed three phylogenetic analyses that differ in the scoring of the terminal taxon from the Lameta Formation. The analysis that only includes the skull of *Jainemys pisdurensis* resolves this species to be located within Kurmademydini as sister to *Sankuchemys sethnai*, while the other two analyses place the shell only taxon or the composite taxon consisting of the skull and shell in an unresolved polytomy at the base of Kurmademydini (Fig. 7). The shell characters that support the unresolved placement within Kurmademydini are the greater midline length of the pectoral than the humeral and the convex lateral margins of the posterior plastron lobe. The cranial character that support placement within Kurmademydini, on the other hand, are the parietal/pterygoid contact lateral to the sulcus palatinopterygoideus and the short rostral margin of the basisphenoid, while placement as sister to *Sankuchemys sethnai* is supported by the narrow contribution of the palatine to the upper triturating surfaces. Unfortunately, of the long number of cranial or postcranial characters that support the monophyly of Bothremydidae, none are preserved in *Jainemys pisdurensis*. However, of the long list of characters that diagnose crown podocnemidids, none are present or preserved in *Jainemys pisdurensis* as well. So, while only a few characters support the placement of *Jainemys pisdurensis* as a kurmademydine, none contradict this hypothesis. Incidentally, the most parsimonious placement of *Jainemys pisdurensis*, the shell material, or the composite taxon within Podocnemidinura (sensu Gaffney et al., 2011) is at its very base, but it is less parsimonious by 4, 5, and 9 steps, respectively. Last, not least, a notable biogeographic signal arises from our analysis, as all turtles from the Late Cretaceous of India are grouped into a clade. We, therefore, have confidence in these results.

The fact that the isolated shell is not resolved in the same place as the cranium is not surprising, given that *Sankuchemys sethnai* is not known from shell material. Our phylogenetic analysis does not provide any evidence for or against the placement of these two terminals on different parts of the tree or for or against the referral of the Lameta shell material to *Jainemys pisdurensis*. Our analysis therefore supports the referral of all postcranial material from the Lameta Formation to Kurmademydini indet. (see above).

The realization that all known Late Cretaceous turtles from India form a clade has immediate implications regarding the biogeographic evolution of pelomedusoids and the paleoecology of kurmademydines. First, the newly established absence of podocnemidids in the Late Cretaceous of India simplifies the biogeographic evolution that clade, as its earliest Cretaceous representatives are thereby restricted to South America, only demanding dispersal to Africa in the Cretaceous (Ferreira et al., 2018). Second, although multiple clades of bothremydid turtles have a wide distribution across the globe (Gaffney, Tong & Meylan, 2006; Ferreira et al., 2018), the clade Kurmademydini is restricted to India, which implies an endemic developed during the Late Cretaceous. Third, this endemic development supports the notion that kurmademydine turtles were inhabitants of fresh water aquatic environments and did not disperse easily across oceanic barriers, in contrast to several other clades of bothremydids. This hypothesis is furthermore supported by the depositional environment in which they are found (see “Geological Settings”).

## Conclusions

*Jainemys pisdurensis* comb. nov. is a valid species of kurmademydine turtle that is based on a partial cranium recovered from the Late Cretaceous (Maastrichtian) Lameta Formation of Pisdura, Maharashtra, India. This increases the known biodiversity of kurmademydine turtles to three, all of which are known from Late Cretaceous (Maastrichtian) sediments exposed across India. Postcranial material from the Lameta Formation of Dongargaon, Maharashtra, which had originally referred to *Jainemys pisdurensis*, can only be diagnosed as Kurmademydini indet. The recognition of a clade of bothremydid turtles unique to India suggests that this continent was isolated from the rest of Gondwana by the Late Cretaceous (Maastrichtian).

## Supplemental Information

10.7717/peerj.9330/supp-1 Supplemental Information 1 List of changes to the matrix of Ferreira et al. (2018).

10.7717/peerj.9330/supp-2 Supplemental Information 2 Character taxon matrix used in phylogenetic analysis in nexus format, including full character list and character state definitions.

10.7717/peerj.9330/supp-3 Supplemental Information 3 A folder that contains all trees in EMF format resulting from the three primary analyses.

## Institutional Abbreviations

ISI: Indian Statistical Institute, Kolkata, India

## Additional Information and Declarations

### Data Availability

The following information was supplied regarding data availability: All specimens described herein are available for study at the Indian Statistical Institute, Kolkata, India (ISI): catalog numbers ISI R185, ISI R186, ISI R187, ISI R188, ISI R189, ISI R190, ISI R191, ISI R192, ISI R193, and ISI R200. The primary data is available in a character/taxon matrix in File S2.

### New Species Registration

The following information was supplied regarding the registration of a newly described species: Publication LSID: urn:lsid:zoobank.org:pub:4842CB09-49CA-4C53-A298-32568EDE1FFF. *Jainemys* LSID: urn:lsid:zoobank.org:act:7C288063-2F1A-4A45-BCD6-5EB95CFAEC8E.

## References

[ref-1] Ambwani K, Sahni A, Kar RK, Dutta D (2003). Oldest known nonmarine diatoms (*Aulacoseira*) from the uppermost Cretaceous Deccan Intertrappean beds and Lameta Formation of India. Revue de Micropaléontologie.

[ref-2] Baur G (1891). Notes on some little known American fossil tortoises. Proceedings of the Academy of Natural Sciences of Philadelphia.

[ref-3] Bona P, de la Fuente MS (2005). Phylogenetic and paleobiogeographic implications of *Yaminuechelys maior* (Staesche, 1929) new comb., a large long-necked chelid turtle from the early Paleocene of Patagonia. Argentina Journal of Vertebrate Paleontology.

[ref-4] Brookfield ME, Sahni A (1987). Palaeoenvironment of the Lameta Beds (Late Cretaceous) at Jabalpur, Madhya Pradesh, India: soil and biotas of a semi-arid alluvial plain. Cretaceous Research.

[ref-5] Cadena EA (2011). Potential earliest record of podocnemidoid turtles from the Early Cretaceous (Valanginian) of Colombia. Journal of Paleontology.

[ref-6] Carter HJ (1852). Geology of the Island of Bombay. Journal of the Bombay Branch of the Royal Asiatic Society.

[ref-7] Chanda SK (1963a). Cementation and diagenesis of the Lameta Beds, Lametaghat. India Journal of Sedimentary Research.

[ref-8] Chanda SK (1963b). Petrology and origin of Lameta sandstone, Lameta Ghat, Jabalpur, M.P., India. Proceedings of the National Institute of Sciences of India.

[ref-9] Chanda SK (1965). Further notes on the origin of Lameta Beds, Jabalpur, M.P.. Science and Culture.

[ref-10] Chanda SK (1967). Petrogenesis of the calcareous constituents of the Lameta Group around Jabalpur, M.P., India. Journal of Sedimentary Research.

[ref-11] Chanda SK, Bhattacharya A (1966). A re-evaluation of the stratigraphy of the Lameta-Jabalpur contact around Jabalpur, M.P.. Journal of the Geological Society of India.

[ref-12] Cope ED (1865). Third contribution to the herpetology of tropical America. Proceedings of the Academy of Natural Sciences of Philadelphia.

[ref-13] de Broin F (1988). Les tortues et le Gondwana. Examen des rapports entre le fractionnement du Gondwana au Crétacé et la dispersion géographique des tortues pleurodires à partir du Crétacé. Studia Palaeocheloniologica.

[ref-14] Fernández MS, Khosla A (2015). Parataxonomic review of the Upper Cretaceous dinosaur eggshells belonging to the oofamily Megaloolithidae from India and Argentina. Historical Biology.

[ref-15] Ferreira GS, Bronzati M, Langer MC, Sterli J (2018). Phylogeny, biogeography and diversification patterns of side-necked turtles (Testudines: Pleurodira). Royal Society Open Science.

[ref-16] França MAG, Langer MC (2005). A new freshwater turtle (Reptilia, Pleurodira, Podocnemidae) from the Upper cretaceous (Maastrichtian) of Minas Gerais, Brazil. Geodiversitas.

[ref-17] Gaffney ES, Chatterjee S, Rudra DK (2001). *Kurmademys*, a new side-necked turtle (Pelomedusoides: Bothremydidae) from the Late Cretaceous of India. American Museum Novitates.

[ref-18] Gaffney ES, Sahni A, Schleich H, Singh SD, Srivastava R (2003). *Sankuchemys*, a new side-necked turtle (Pelomedusoides: Bothremydidae) from the Late Cretaceous of India. American Museum Novitates.

[ref-19] Gaffney ES, Tong H, Meylan PA (2006). Evolution of the side-necked turtles: the families Bothremydidae, Euraxemydidae, and Araripemydidae. Bulletin of the American Museum of Natural History.

[ref-20] Gaffney ES, Meylan PA, Wood RC, Simons E, De Almeida Campos D (2011). Evolution of the side-necked turtles: the family podocnemididae. Bulletin of the American Museum of Natural History.

[ref-21] Gaffney ES, Krause DW (2011). *Sokatra*, a new side-necked turtle (Late Cretaceous, Madagascar) and the diversification of the main groups of Pelomedusoides. American Museum Novitates.

[ref-22] Gaffney ES, Krause DW, Zalmout IS (2009). *Kinkonychelys*, a new side-necked turtle (Pelomedusoides: Bothremydidae) from the Late Cretaceous of Madagascar. American Museum Novitates.

[ref-23] Georgalis GL, Velitzelos E, Velitzelos DE, Kear BP, Brinkman DB, Holroyd PA, Gardner JH (2013). *Nostimochelone lampra* gen. et sp. nov., an enigmatic new podocnemidoidean turtle from the Early Miocene of Northern Greece. Fossil European Sea Turtles: A Historical Perspective.

[ref-24] Goloboff PA, Farris JS, Nixon K (2008). TNT: a free program for phylogenetic analysis. Cladistics.

[ref-25] Goloboff PA, Torres A, Arias JS (2018). Weighted parsimony outperforms other methods of phylogenetic inference under models appropriate for morphology. Cladistics.

[ref-26] Jain SL (1977). A new fossil pelomedusid turtle from the Upper Cretaceous Pisdura sediments, central India. Journal of the Palaeontological Society of India.

[ref-27] Jain SL (1986). New pelomedusid turtle (Pleurodira: Chelonia) remains from Lameta Formation (Maastrichtian) at Dongargaon, central India, and a review of Pelomedusids from India. Journal of the Palaeontological Society of India.

[ref-28] Jain SL, Gillet DD, Lockley MG (1989). Recent dinosaur discoveries in India, including eggshells, nests and coprolites. Dinosaur Tracks and Traces.

[ref-29] Jain SL, Bandyopadhyay S (1997). New titanosaurid (Dinosauria: Sauropoda) from the Late Cretaceous of central India. Journal of Vertebrate Paleontology.

[ref-30] Jain SL, Sahni A, Maheshwari HK (1983). Some upper Cretaceous vertebrates from central India and their palaeogeographical implications. Cretaceous of India, Indian Association of Palynostratigraphers Symposium.

[ref-31] Kapur VV, Khosla A (2019). Faunal elements from the Deccan volcano-sedimentary sequences of India: a reappraisal of biostratigraphic, palaeoecologic, and palaeobiogeographic aspects. Geological Journal.

[ref-32] Khosla A, Sahni A (1995). Parataxonomic classification of Late Cretaceous dinosaur eggshells from India. Journal of the Palaeontological Society of India.

[ref-33] Khosla A, Verma O (2015). Paleobiota from the Deccan volcano-sedimentary sequences of India: paleoenvironments, age and paleobiogeographic implications. Historical Biology.

[ref-34] Khosla A, Chin K, Alimohammadin H, Dutta D (2015). Ostracods, plant tissues, and other inclusions in coprolites from the Late Cretaceous Lameta Formation at Pisdura, India: taphonomical and palaeoecological implications. Palaeogeography, Palaeoclimatology,Palaeoecology.

[ref-35] Khosla A, Chin K, Verma O, Alimohammadin H, Dutta D (2016). Paleobiogeographical and paleoenvironmental implications of the freshwater Late Cretaceous ostracods, charophytes and distinctive residues from coprolites of the Lameta Formation at Pisdura, Chandrapur District (Maharashtra), Central India. New Mexico Museum of Natural History and Science Bulletin.

[ref-36] Klein IT (1760). Klassification und kurze Geschichte der vierfüßigen Thiere (translation by F.D. Behn).

[ref-37] Lydekker R (1890). Note on certain vertebrate remains from the Nagpur District. Records of the Geological Survey of India.

[ref-38] Mohabey DM (1987). Juvenile sauropod dinosaur from Upper Cretaceous Lameta Formation of Panchmahals District, Gujarat. India Journal of the Geological Society of India.

[ref-39] Mohabey DM (1996). Depositional environments of Lameta Formation (Late Cretaceous) of Nand-Dongargaon inland basin, Maharashtra: the fossil and lithological evidences. Memoir Geological Society of India.

[ref-40] Mohabey DM, Head JJ, Wilson JA (2011). A new species of the snake *Madtsoia* from the Upper Cretaceous of India and its paleobiogeographic implications. Journal of Vertebrate Paleontology.

[ref-41] Mohabey DM, Samant B (2003). Floral remains from Late Cretaceous faecal mass of sauropods from Central India: implications to their diet and habitat. Gondwana Geological Magazine.

[ref-42] Mohabey DM, Udhoji SG (1996). Fauna and flora from Late Cretaceous (Maestrichtian) non-marine Lameta sediments associated with Deccan volcanic episode, Maharashtra: its relevance to the K-T boundary problem, palaeoenvironment and palaeogeography. Gondwana Geological Magazine.

[ref-43] Mohabey DM, Udhoji SG (2000). Vertebrate fauna of Late Cretaceous dinosaur-bearing Lameta Formation of Nand-Dongargaon inland basin, Maharashtra: palaeoenvironment and K-T boundary implications. Memoirs of the Geological Society of India.

[ref-44] Mohabey DM, Udhoji SG, Verma KK (1993). Palaeontological and sedimentological observations of nonmarine Lameta Formation (Upper Cretaceous) of Maharashtra, India: their palaeoecological and palaeoenvironmental significance. Palaeogeoraphy, Palaeoclimatology, Palaeoecology.

[ref-45] Pérez-García A, Ortega F, Murelaga X (2012). A new genus of Bothremydidae (Chelonii, Pleurodira) in the Cretaceous of Southwestern Europe. Geobios.

[ref-46] Prasad GVR, Cappetta H (1993). Late Cretaceous selachians from India and the age of Deccan Traps. Paleontology.

[ref-47] Singh IB (1981). Palaeoenvironment and palaeogeography of Lameta Group sediments (Late Cretaceous) in Jabalpur area. India Journal of the Palaeontological Society of India.

[ref-48] Singh IB, Srivastava HK (1981). Lithostratigraphy of Bagh Beds and its correlations with Lameta beds. Journal of the Palaeontological Society of India.

[ref-49] Srivastava A, Mankar RS (2015). *Megaloolithus* dinosaur nest from Lameta succession of Salbardi area, districts Amravati, Maharashtra and Betul, Madhya Pradesh. Journal of the Geological Society of India.

[ref-50] Suárez JM (1969). Um quelônio da Formação Baurú: Anais do XXIII Congresso Brasileiro de Geologia.

[ref-51] Swinton WE (1939). A new fresh-water tortoise from Burma. Records of the Geological Survey of India.

[ref-52] Sykes C (1851). On a fossil fish from the table land of the Deccan, in the Peninsula of India, with a description of specimens by P.M.G. Egerton. Quarterly Journal of the Geological Society of London.

[ref-53] Tandon SK, Sood A, Andrews JE, Dennis PF (1995). Palaeoenvironment of the dinosaur bearing Lameta Beds (Maastrichtian), Narmada Valley, Central India. Palaeogeography, Palaeoclimatology, Palaeoecology.

[ref-54] Tandon SK, Verma VK, Jhingran V, Sood A, Kumar S, Kohli RP, Mittal S (1990). The Lameta Beds of Jabalpur, Central India: deposits of fluvial and pedogenically modified semi-arid pre-palustrine flat systems.

[ref-55] Rhodin AGJ, Iverson JB, Bour R, Fritz U, Georges A, Shaffer HB, Van Dijk PP, Turtle Taxonomy Working Group (TTWG) (2017). Turtles of the world: annotated checklist and atlas of taxonomy, synonymy, distribution, and conservation status. Chelonian Research Monographs.

[ref-56] von Huene F, Matley CA (1933). The Cretaceous Saurischia and Ornithischia of the central provinces of India. Memoirs of the Geological Survey of India, Palaeontologica Indica: New Series.

[ref-57] Weems RE, Knight JL, Brinkman DB, Holroyd PA, Gardner JH (2013). A new species of *Bairdemys* (Pelomedusoides: Podocnemididae) from the Oligocene (Early Chattian) Chandler Bridge Formation of South Carolina, USA, and its paleobiogeographic implications for the genus. Morphology and Evolution of Turtles.

[ref-58] Williams EE (1953). Fossils and the distribution of chelyid turtles, *Hydraspis leithii* (Carter) in the Eocene of India is a pelomedusid. Breviora.

[ref-59] Wilson JA, Upchurch P (2003). A revision of *Titanosaurus* Lydekker (Dinosauria—Sauropoda), the first dinosaur genus with a ‘Gondwanan’ distribution. Journal of Systematic Palaeontology.

[ref-60] Wood RC (1970). A review of the fossil Pelomedusidae (Testudines, Pleurodira) of Asia. Breviora.

[ref-61] Wood RC (1985). Evolution of the pelomedusid turtles. Studia Palaeocheloniologica.

[ref-62] Woodward AS (1908). On some fish remains from the Lameta Beds at Dongargaon, Central Province. Memoirs of the Geological Survey of India, Paleontologica Indica New Series.

