# Supplementary material for: A revision of the pelomedusoid turtle Jainemys pisdurensis from the Late Cretaceous (Maastrichtian) Lameta Formation of India
Source: PeerJ. 2020 Jun 22;8:e9330. doi: 10.7717/peerj.9330 (PMC7316078; doi:10.7717/peerj.9330)
Supplement: Supplemental Information 1 [file peerj-08-9330-s001.docx]

**Supplemental File S1: List of changes to the matrix of *Ferreira et al.* (*2018*)**

*Kinkonychelys rogersi*: The following changes in the scoring of this taxon were implemented based on the descriptions provided by *Gaffney, Krause & Zalmout* (*2009*).

5: preorbital skull width: 1, not 0

14: lower temporal emargination: 1, not ?

23: parietal/jugal contact: 0, not ?

40: postorbital size: 0, not ?

55: posterior lingual ridge: 0, not 1

57: triturating surfaces: 1, not ?

58: medial expansion of triturating surfaces: 0, not ?

63: maxilla/quadratojugal contact: 0, not ?

64: maxilla/quadrate contact: 0, not ?

73: apertura narium interna: ?, not 0

76: secondary palate: -, not ?

79: dorsal process of palatine: 0, not ?

90: ventral outline of quadrate: ? not 1

116: basioccipital tubera width: 0, not ?

123: fenestra postotica: 0, not 1

126: processus paroccipitalis: ?, not 1

*Kurmademys kallamedensis*: The following changes in the scoring of this taxon were implemented based on the descriptions provided by *Gaffney, Chatterjee & Rudra* (*2001*) and *Gaffney, Tong & Meylan* (*2006*).

4: midline contact of nasals: -, not ?

9: interorbital groove: 0, not ?

12: prefrontal/frontal contact: 0, not ?

26: parietal/pterygoid contact: 0, not ?

55: posterior lingual ridge of maxilla: 0, not 1

58: medial expansion of triturating surface: 0, not ?

79: dorsal process of palatine: 0, not ?

80: dorsal process reaching septum: 1, not 0

85: incisura columella auris ridge: -, not 1

110: condylus occipitalis: ?, not 0

116: basioccipital tubera: 0, not ?

129: rostral margin of basisphenoid: 1, ot 2

133: splenial: 1, not ?

135: dentary angle: 0, not ?

138: accessory ridge of dentary: 0, not ?

145: coronoid in triturating surface: 1, not ?

177: neural/nuchal contact; 0, not ?

179: keeled neurals: 0, not ?

184: suprapygal/peripheral 10 contact: 1, not ?

207: vertebral 1 shape: ?, not 1

217: anterior lobe length: ?, not 0

219: plastral lobe length: 1, not 0

*Sankuchemys sethnai*: The following changes in the scoring of this taxon were implemented based on the descriptions provided by *Gaffney et al.* (*2003*).

2: dorsal margin apertura narium externa: 0, not ?

4: midline contact of nasals: -, not ?

9: interorbital groove: 0, not ?

12: prefrontal/frontal contact: 0, not ?

15: quadratojugal: 1, not ?

23: parietal/jugal contact: 0, not ?

32: jugal/quadrate contact: 0, not ?

35: caudal projection of squamosal: 0, not ?

36: caudoventral vertical flange of squamosal: 0, not ?

37: lateral tubercle of squamosal: 0, not ?

38: lateral surface of squamosal: 0, not ?

54: labial ridge of maxilla: 0, not ?

57: triturating surface shape: 1, not ?

58: medial expansion of triturating surface: 0, not ?

59: triturating surface convexity: 0, not ?

66: vomer/premaxilla contact: 1, not ?

71: foramen palatinum posterius: 1, not 0

73: apertura narium interna: 0, not ?

74: dorsally arched palate: 0, not ?

75: palatine in triturating surface: 0, not 1

101: fpcci: 2, not 0

103: supraoccipital exposure: 0, not ?

107: supraoccipital on otic chamber: 0, not ?

110: condylus occipitalis: 1, not ?

115: basioccipital length: 0, not 1

117: horizontal occipital shelf: 0, not ?

120: foramen stapedio-temporale: 0, not ?

121: foramen stapedio-temporale and trigemini: 0, not ?

129: rostal margin of basisphenoid: 1, not ?

*Sokatra antitra*: The following changes in the scoring of this taxon were implemented based on the descriptions provided by *Gaffney & Krause* (*2011*).

18: depth of upper temporal emargination: 2 instead of ?

20: lateral edges of parietals: 1, not ?

25: parietal pterygoid contact at base of processus trochlearis pterygoidei: 0, not ?

40: size of postorbital: 0, not 1

57: shape of triturating surface: 0, not ?

58: medial expansion of triturating surface: 0, not ?

62: exposure of maxilla in orbit: 1, not 0

82: incisura columella auris: 1, not 2

83: stapes in canal: 1, not 0

85: trough on incisura columellae auris ridge: -, not ?

103: exposure of supraoccipital on skull roof: 1, not ?

104: length of crista supraoccipitalis: ?, not 0

109: ventral process of exoccipital: 0, not ?

110: condylus occipitalis: ?, not 0

122: ventral covering of processus interfenestralis: ?, not 1

124: shape of fenestra postotica: ?, not 0

126: processus paroccipitalis: ? not 0

136: high lingual ridge of dentary: 1, not 0

142: triturating surfaces: 1, not ?

145: participation of coronoid in triturating surfaces: 1, not ?
